# Unusual Long Survival with a Giant Invasive Pheochromocytoma of an Incompatible Patient

**DOI:** 10.7759/cureus.2319

**Published:** 2018-03-13

**Authors:** Asli Nar

**Affiliations:** 1 Department of Endocrinology and Metabolism, Baskent University Faculty of Medicine, Ankara

**Keywords:** malignant pheochromocytoma, survival, 3-methoxytyramine, radionuclide therapy

## Abstract

Pheochromocytomas (PHEOs) are rare neuroendocrine tumors and about 2-13% of PHEOs are malignant. Predicting malignancy in PHEO cases with invasion but without metastasis is still controversial in the literature. This study presents an unusual long survival with a giant invasive PHEO in an incompatible patient and a review of the literature. In 1989, a 23-year-old female patient was operated for a giant adrenal mass with a pathological final diagnosis of PHEO. Information to the patient’s family was provided about the short life span of the patient in the postoperative period because the tumor could not be totally resected. The patient started using regular antihypertensive drugs only after 1994. In 1994, 3700 mBq 131-I-metaiodobenzylguanidine (MIBG) treatment was given. Since then, no specific treatment was administered for PHEO due to patient incompatibility. She was diagnosed with type 2 diabetes mellitus at the age of 40 years and had a cerebrovascular accident due to hypertension at the age of 42. New abdominal computed tomography (CT) showed a right-sided 75 x 37 mm irregular and heterogeneous mass lesion extending inferiorly from the diaphragmatic crus level located in the right adrenal locus compatible with local recurrence. There was no I-123-MIBG uptake. She refused to have advanced workup and further treatment options. Malignant PHEOs reduce overall survival as a consequence of excessive catecholamine release, large tumor burden, and malignancy-related complications. Currently, the treatment of a malignant PHEO still has difficulties for both patients and doctors. Main treatment options for malignant PHEOs are primarily surgical excision. The effect of radionuclide therapy on survival time still remains to be determined. Efforts should be made to identify clinical, biochemical, and pathological criteria for malignancy and to develop new therapies in these patients with malignancy. The clinical course of malignant PHEOs is remarkably variable. Disease-specific survival rate changes from 58 to 88.1% at five years in the literature. Recent discoveries have enhanced new options for treatment, from radionuclide therapy and targeted molecular therapy to immunotherapy. A multidisciplinary approach is needed to individualize treatment in patients with malignant and invasive PHEO.

## Introduction

Pheochromocytomas (PHEOs) are rare neuroendocrine tumors of the chromaffin cells manifested by catecholamine hypersecretion in the adrenal medulla. PHEOs (in Greek, pheos means dusky, chroma means color, and cytoma means tumor. PHEOs = dusky-colored tumors) were reported to occur sporadically or genetically (multiple endocrine neoplasia type 2, neurofibromatosis type 1, Von Hippel-Lindau disease (VHL), mutations in succinate dehydrogenase subunit B (SDHB)) [1].

A recent study conducted at the Mayo Clinic reported that the incidence rate of malignant PHEO and paraganglioma was 8.3% (272 of 3,280 patients) between the years 1960 and 2016 [2]. Since the growth rate of tumor varies from patient to patient and since it has a rare incidence, it is not possible to determine the actual prognosis in these patients. In addition, the definition of malignancy is variable for PHEO and this condition has led to different reports of mortality rates in the literature. According to the World Health Organization (WHO), in order to be able to diagnose malignant PHEO, it is necessary to have metastasis at nonchromaffin sites [3]. However, predicting malignancy in PHEO cases with invasion but without metastasis is still controversial in the literature [2]. Therefore new clinical, biochemical, and pathological markers are needed to determine malignancy in PHEO.

The main treatment options for malignant PHEO are primarily surgical excision, then systemic chemotherapy, radiopharmaceutical therapy, and targeted molecular therapy [1].

This study describes a rare case of an incompatible patient with a giant invasive PHEO who refused optimum treatment and had long survival. We also performed a literature review based on this case of invasive PHEO.

## Case presentation

A 23-year-old woman living in a rural area of Turkey presented to multiple doctors with the complaints of headache, hyperhydrosis, and abdominal pain attacks as fainting crises in 1988. She did not recall a measurement of her blood pressure during those doctor visits. After one year of intense crises, one doctor discovered attacks of hypertension, as high as 220/140 mmHg. Another doctor in Istanbul found a giant right adrenal mass in this patient. In 1989, she underwent subtotal excision of the tumor, right adrenalectomy, and right nephrectomy, but she had cardiac arrest several times during the operation and had to be resuscitated. The postoperative pathological examination showed PHEO with extensive degenerative and congestive changes in the tumor, in addition to invasion of the adrenal capsule and right kidney (tumor weight was over 1000 g). Information to the patient’s family was provided about the short life span of the patient in the postoperative period because the tumor could not be totally resected. The patient did not go to further doctor checkups as the patient and her family were waiting for her so-called inevitable death. Until 1994, antihypertensive agents were used irregularly by the patient, but then antihypertensive agents were given regularly for high blood pressure. In 1994, 3700 mBq I-131- metaiodobenzylguanidine (MIBG) treatment was given in Germany. Since then, no specific treatment was administered for PHEO. She was diagnosed with type 2 diabetes mellitus since she was 40 years old. She had a cerebrovascular accident due to hypertension at the age of 42 with sequelae of speech problems. The family history was negative for PHEO.

She applied to our endocrinology clinic for the regulation of her medications in July 2017. Her current medications included phenoxybenzamine 1 × 10 mg, doxazosin 1 × 4 mg, bisoprolol 1 × 5 mg, amlodipine 1 × 10 mg, metformin 2 × 1000 mg, and gliclazide mr 1 × 30 mg.

The following were detected as abnormal routine laboratory tests: anemia - Hb: 10.3 g/dl, creatinine - 1.31 mg/dl, HbA1c - 6.8%, LDL cholesterol - 137.5 mg/dl. The plasma free metanephrine was 1.07 nmol/L (0.08-0.51), plasma free normetanephrine was 21.70 nmol/L (0.12-1.18), plasma 3-methoxytyramine (MT) was 0.36 nmol/L (0.00-0.17), and chromogranin A level was 699.80 ng/ml (<94).

**Figure 1:**
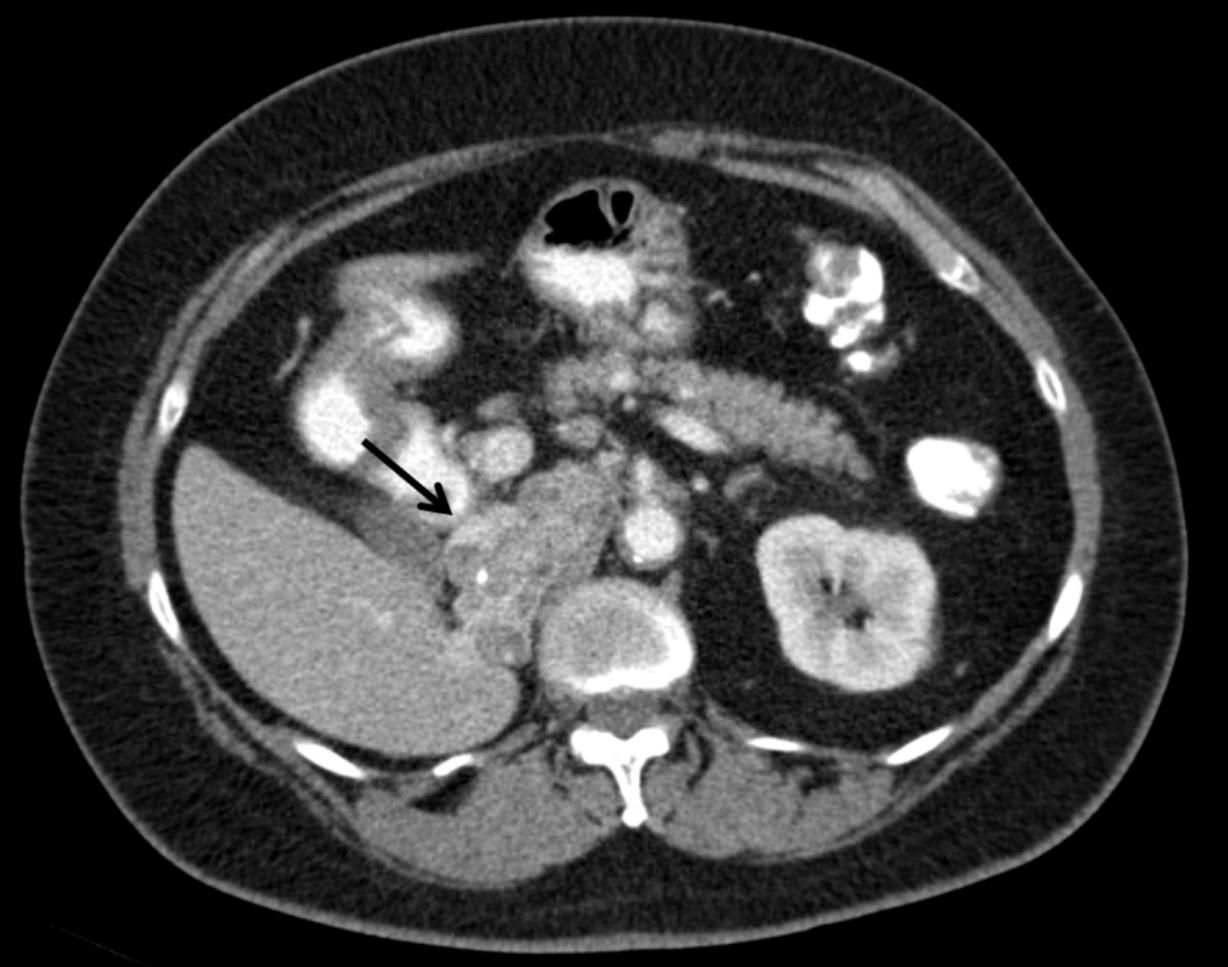
Transverse plane of computed tomography scan revealing invasive pheochromocytoma (black arrow)

**Figure 2:**
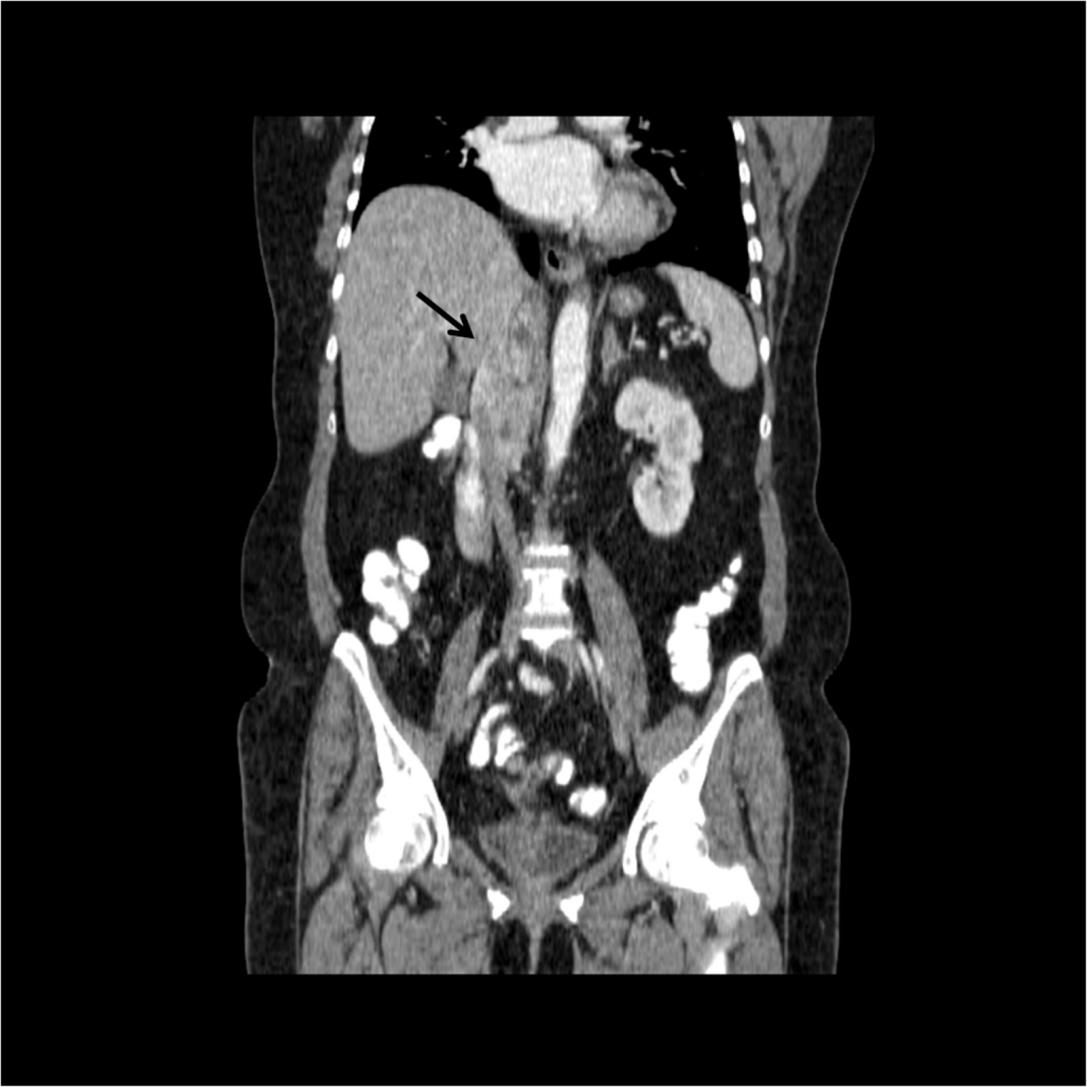
Right-sided 75 x 37 mm irregular and heterogeneous mass lesion extending inferiorly from the diaphragmatic crus level, invasive to vena cava inferior and right lobe of the liver (black arrow)

An abdominal computed tomography (CT) scan showed a right-sided 75 x 37 mm irregular and heterogeneous mass lesion extending inferiorly from the diaphragmatic crus level located in the right adrenal locus compatible with local recurrence (Figure 1, Figure 2).

The lesion showed heterogeneous enhancement with occasional cystic necrotic areas and a calcified focus. It was observed that the mass was invasive to the vena cava inferior and showed nodular invasion approximately 1 cm in diameter in the subcapsular area in the inferior segment 6 level of the right lobe of the liver. The appearances that suggest the tumor thrombus is remarkable in the vena cava inferior. Metastasis was not detected in the patient's thorax computerized tomography scan.

After a multidisciplinary discussion, an extensive surgery and then chemotherapy were recommended to the patient. The patient did not want to be reoperated on and refused to have advanced workup including genetic studies. 131-I-MIBG treatment was planned because our patient did not accept surgery and chemotherapy. For this reason, screening with I-123-MIBG was performed. Unfortunately, there was no I-123-MIBG uptake in our patient. 68Ga-DOTA(0)-Tyr(3)-octreotate (68Ga-DOTATATE) positron emission tomography (PET) /CT scanning was planned but the patient refused it.

## Discussion

Here we present a 52-year-old female patient with a challenging case of PHEO. The patient underwent subtotal tumor resection in 1989 when she was 23 years old. In 1994 she received I-131-MIBG treatment. Our patient was alive for 29 years despite a number of hypertensive complications and tumor burden.

In the literature, malignant PHEOs show variable growth rate and thus survival. Depending on the different definitions of malignancy in the literature and also considering the known sparsity of malignant PHEO, five-year survival rates range from 20 to 70% in the studies [2,4]. Excessive catecholamine release in metastatic and bulky PHEO increases the morbidity and mortality rates especially by causing cardiovascular complications. In the literature, hypertensive crisis, cardiac dysfunction, tumor burden, and tumor progression are reported as causes of death in malignant PHEO [2].

The WHO stated that malignant PHEO is diagnosed only by the documented presence of metastases to tissues where chromaffin cells are not present (like lymph nodes, lung, bones, and liver) and less emphasis was placed on local invasion [3]. However, in 2007, the Armed Forces Institute of Pathology Fascicle Tumours of the Adrenal Glands and Extra-adrenal Paraganglia defined malignancy as “extensive local invasion or documentation of metastases” [5]. Metastases are most frequently observed in lymph nodes (80%), skeleton (72%), liver (50%), and lung (50%) in PHEO patients [1].

About 50% of patients with metastatic disease have particular hereditary germline mutations [6]. Determining a genetic mutation may prognosticate the disease and predict the ways of treatment. Genes that play a role in PHEO tumorigenesis are involved in pseudohypoxic and kinase receptor signaling processes causing dysregulation of cellular principal metabolic pathways [6]. Genetic testing should be evaluated in PHEO patients with a positive family history, syndromic features, and multifocal, bilateral, or metastatic disease [7]. In the absence of a syndromic, familial, or metastatic presentation, selection of genes for testing may be guided by biochemical phenotype [7]. Generally, if there is a known PHEO family history and a related mutation, only the particular mutation should be investigated.

Biochemical tests should be performed after clinical suspicion to ensure diagnosis. Samples for plasma and urinary catecholamines and metanephrines must be taken under appropriate conditions. The Endocrine Society Clinical Practice Guideline for Pheochromocytoma and Paraganglioma recommends that initial biochemical testing should be plasma free metanephrines or urinary fractionated metanephrines [7]. Lenders and Eisenhofer suggest that plasma free metanephrines combined with 3-MT, a metabolite of dopamine, offer a slightly higher sensitivity (99%) than that of urinary metanephrines (95%) in the diagnosis of PHEO [8]. They also claimed that the upper cut-off value for plasma 3-MT is 0.10 nmol/L. Although plasma metanephrines and 3-MT are slightly higher in males than in females, no gender-specific cut-off reference values are required in clinical care.

3-MT is thought to be a novel malignancy marker nowadays in addition to other malignancy risk factors like SDHB mutation, tumor diameter greater than 5 cm, and tumors in non-chromaffin locations [9]. 3-MT levels were found to be 4.7 times higher in patients with malignant (metastatic) PHEO than in patients without metastases [9]. For the first time in this study, plasma 3-MT has been shown to be a more sensitive biomarker of tumoral dopamine secretion and metastasis than plasma or urinary dopamine levels. Nevertheless, Eisenhofer et al. also found overlap in distributions of MT in some patients with and without metastases.

In our patient with invasive PHEO, 3-MT was not detected at the expected levels of malignancy. However, the plasma free normetanephrine level was found above the level stated in the study of Eisenhofer et al. (Mean plasma free normetanephrine level for metastatic PHEO was 5.51 (4.10-7.40) nmol/L vs 21.70 nmol/L in our patient [9]. The fact that 3-MT does not rise as in metastatic cases suggests that there may not be metastatic spread despite the presence of a giant tumor in our patient.

Thus, until a more reliable metastatic biomarker is found than 3-MT, this measurement may nevertheless give predictive information about the size, metastasis presence, and SDHB mutation status of PHEO.

Once the PHEO is biochemically confirmed, imaging with CT or magnetic resonance imaging (MRI) is then performed. The Endocrine Society Clinical Practice Guideline for Pheochromocytoma and Paraganglioma suggests CT rather than MRI as the first-choice imaging modality because of its excellent spatial resolution for the thorax, abdomen, and pelvis [7].

Functional imaging is recommended in the presence of an adrenal mass over 5 cm and in the presence of a hereditary syndrome [7]. 123-I-MIBG, 2-deoxy-2-[fluorine-18]fluoro-D-glucose (18F-FDG) PET/CT, 111-In-DTPA-octreotide (Octreoscan), 18F-FDOPA-PET/CT, and 68Ga-DOTATATE PET/CT are used to investigate the presence of metastasis and extent of the tumor. Using different functional imaging modalities in the same patient helps to understand the real extention of the disease but this is not a practical method and, in general, all mentioned radiopharmaceuticals are not available in the same hospital. The Endocrine Society Clinical Practice Guideline for Pheochromocytoma and Paraganglioma suggests the use of 18F-FDG PET/CT scanning in patients with metastatic disease since 18F-FDG PET/CT seems to be a superior imaging modality over I-123-MIBG scintigraphy in patients with known metastatic PHEOs and paragangliomas [7].

The Endocrine Society Clinical Practice Guideline for Pheochromocytoma and Paraganglioma suggests measuring plasma or urine levels of metanephrines after surgery to diagnose persistent disease and lifelong annual biochemical testing to assess for recurrent or metastatic disease [7].

Extensive local invasion to adjacent tissues does not always indicate higher risk towards development of metastases like our case [1]. This limitation indicates that the studies were prone to selection bias, were fragmented and descriptive, thereby potentially falsely lowering the mortality risk [4].

In a study conducted by Hamidi et al. on 97 patients with malignant PHEO who presented to the Mayo Clinic from 1960 to 2016, the five-year overall survival rate was 79.2%, the 10-year overall survival rate was 64.2% for PHEO, and the 15-year overall survival was 65.4% [2]. The median disease-specific survival was 33.7 years. They defined malignant disease in accordance with the 2004 WHO criteria [3]. The disease-specific survival rates were 88.1% at five years, 77.9% at 10 years, and 71.8% at 15 years. The overall and disease-specific survival rates were similar for patients with metastatic PHEO and paraganglioma (PGL) (P= 0.22 and P= 0.87). Fourteen of the PHEO patients were considered rapidly progressive because of median survival of 1.6 years from diagnosis time. Hamidi et al. suggested that increased mortality is associated with male sex, older age at diagnosis, synchronous metastases, larger tumor size, elevated dopamine, and not undergoing resection of primary tumor [2].

A cohort study published in 2012 found that 281 malignant PHEO patients showed a five-year survival rate of 58.1% between 1988 and 2009 [10]. This study collected data from 18 state registries that cover about 28% of the US population. Seventy-two percent of the cases were larger than 5 cm in size. A lower overall survival for patients with malignant PHEO correlated with male gender, age higher than 76 years, having a tumor size larger than 5 cm, presenting with distant metastases, not undergoing any surgery, necessitating radiotherapy, and having distant metastasis. Local and regional invasion did not cause increased mortality. They emphasized that long-term survival did not improve over the last two decades [10].

In the meta-analysis carried out by Hamidi in 2017, the five-year mortality rate was found to be 42% (37-48%) in 685 metastatic PHEO patients in 20 studies published between 1992-2016 [4]. A higher mortality was associated with male sex (relative risk (RR) 1.50; 95% confidence interval (CI), 1.11-2.02) and synchronous metastases (RR 2.43; 95% CI, 1.01-5.85). They concluded that their study shows highly variable mortality rates in patients with malignant PHEO mainly because of a variety of metastatic assessments, thereby increasing heterogeneity of the included studies.

The Endocrine Society Clinical Practice Guideline for Pheochromocytoma and Paraganglioma recommends minimally invasive adrenalectomy (e.g. laparoscopic) for most adrenal PHEOs [7]. They recommend open resection for large (eg: >6 cm) or invasive PHEOs to ensure complete tumor resection, to prevent tumor rupture and thus prevent local recurrence.

If curative surgery is not possible, systemic chemotherapy, radiopharmaceutical therapies, targeted molecular therapy, and immunotherapy may be considered in the treatment of malignant PHEO [1].

Cyclophosphamide, vincristine, and dacarbazine (CVD) combination has been the most frequently used systemic chemotherapy [1]. CVD chemotherapy in malignant PHEO was showed to reduce catecholamine levels and tumor mass, but these positive response rates may be overestimated.

I-131-MIBG therapy has demonstrated tumor reduction and disease stabilization in metastatic PHEO. I-131-MIBG binds to the catecholamine reuptake transporter with high affinity on the cell membrane, resulting in radiation-induced cell death [1]. Treatment of malignant PHEO with I-131-MIBG shows partial remission rates of 20%-47% in some studies. Complete and permanent responses are uncommon. Radionuclide therapy offers different results depending on tumor size and spread and appears to be rather palliative than being curative.

In patients with inoperable malignant PHEO that did not have I-123-MIBG uptake, investigators have sought new peptide receptor radionuclide therapy. When the results of lutetium-177 (177LU)-DOTATATE treatment are analyzed, treatment seems to achieve important clinical and biochemical responses with low toxicity and encouraging survival in metastatic PHEO and paragangliomas [6].

The effect of radionuclide therapy on survival time still remains to be determined. For these reasons, there is a need for new systemic treatments to increase the survival of patients with malignant PHEO.

Tyrosine kinase receptor inhibitors have strong anti-tumoral and anti-angiogenic effects. Phase II studies with these agents in malignant PHEO show promising initial results [1]. As immunotherapy, pembrolizumab is a new drug consisting of programmed death ligand receptor-1 (PD-L1) antibody proven to be useful for numerous types of cancer cells. Anti-PD1 inhibitors may represent a novel treatment option for metastatic PHEO refractory to other treatments [1].

Our patient survived so long (29 years) despite the presence of a giant invasive tumor and without proper follow-up and treatment. We believe that I-131 MIBG treatment has been very useful in increasing the survival rate in our patient who received 3700 mBq I-131 MIBG treatment in 1994. Based on this literature review, guideline recommendations, and the clinical presentation of our patient, we may classify our patient as having invasive PHEO. Nevertheless, tumor control and the patient’s condition would be better if the patient went to regular doctor visits and if she did not have the hypertensive cerebrovascular accident.

## Conclusions

PHEOs are rare neuroendocrine tumors of chromaffin cells in the adrenal medulla. These tumors reduce overall survival as a result of excessive catecholamine release, large tumor burden, and malignancy-related complications. Currently, the treatment of malignant PHEO is still difficult for both patients and doctors. However, malignancy criteria have not yet been standardized in the literature. Efforts should be made to identify clinical, biochemical, and pathological criteria for malignancy and to develop new therapies in these patients with malignancy. The clinical course of malignant PHEO is remarkably variable. Recent discoveries have enhanced new options for treatment, from radionuclide therapy and targeted molecular therapy to immunotherapy. A multidisciplinary approach is needed to individualize treatment in patients with malignant and invasive PHEO.
